# 518 Prescribing practices of atypical antipsychotics in the burn ICU

**DOI:** 10.1093/jbcr/irac012.149

**Published:** 2022-03-23

**Authors:** Rita Gayed, Kristen Robinson, Annalise Labatut, Rohit Mittal

**Affiliations:** Grady Health System, Atlanta, Georgia; VA Portland Health Care System, Portland, Oregon; Grady Health System, Atlanta, Georgia; Emory University, Atlanta, Georgia

## Abstract

**Introduction:**

Atypical antipsychotics are commonly used in the management of agitation and delirium in the intensive care unit (ICU). Patients admitted to the burn intensive care unit (BICU) with burns with large total body surface involvement ( >20%) require prolonged mechanical ventilation and prolonged ICU stay, putting them at risk of ICU delirium. Furthermore, patients with burn injuries often have underlying psychiatric conditions, and some can develop new psychiatric disorders secondary to the trauma associated with their burn. Due to these factors many burn patients receive scheduled oral atypical antipsychotics during their ICU stay. The purpose of this study was to retrospectively characterize the prescribing practices of atypical antipsychotics in the BICU.

**Methods:**

This was a single-center, retrospective chart review of adults admitted to the BICU with a burn injury who received scheduled oral atypical antipsychotics. Prescribing patterns in the ICU and on all transitions of care were analyzed. Additionally, the appropriateness of AAP prescribing at discharge was evaluated. AAPs were considered to be appropriately prescribed at discharge if a patient was continuing a home medication, or if psychiatric consult services recommended continuing at discharge.

**Results:**

During the five year study period, 440 adults were admitted to the BICU with a burn injury, 18.2% of which were prescribed an AAP during their ICU course. Of those prescribed an AAP, 28.8% had a documented underlying psychiatric condition. Most patients were male (70%) with an average age of 41 years, and a mean total body surface area burn of 32%. The average ICU length of stay was 32 days. AAPs were primarily used to treat agitation/delirium (72.5% of patients). Quetiapine was the most commonly prescribed AAP. On transfer to stepdown, AAPs were continued in 78.4% of patients. Additionally, 67.7% were discharged on an AAP. Of these patients, continuation was considered appropriate in 54% of patients.

**Conclusions:**

Despite overall lower AAP prescribing in the burn ICU compared to other ICUs, over two thirds of patients initiated on AAPs in the BICU were prescribed AAPs at discharge. AAPs should be evaluated for appropriateness at each transition of care.

